# Case Report: *BRCA1* and *BRCA2* loss in a young man with primary cutaneous extraskeletal osteosarcoma

**DOI:** 10.3389/fonc.2025.1504366

**Published:** 2025-03-04

**Authors:** Wen-Feng Luo, Yu-Hang Hou, Yu-Teng Huang, Jun-Dong Lai, Hui-Shan Jiang, Wei-Liang Wang

**Affiliations:** ^1^ Guangdong Medical University, Zhanjiang, Guangdong, China; ^2^ Yangjiang People’s Hospital affiliated to Guangdong Medical University, Yangjiang, Guangdong, China

**Keywords:** osteosarcoma, primary cutaneous extraskeletal osteosarcoma, soft tissue tumor, BRCA1, BRCA2

## Abstract

**Background:**

Extraskeletal osteosarcoma is an uncommon and high-grade soft tissue malignancy. The incidence is even lower when the skin is the primary site. To the best of our knowledge, the primary cutaneous osteosarcoma has fewer than 30 reported cases worldwide, which with decreased copy number of*BRCA1* and *BRCA2* has never been reported before.

**Case presentation:**

A 28-year-old man was hospitalized for a skin mass on the left shoulder. The histological examination showed a large number of tumor giant cells and fibroblasts, and nuclear division was easy to see. Immunohistochemistry showed positive for CK, EMA, S100, CD34, CK7, Bcl-2, ACTin, and NSE, and negative for Vim, SATB2, CD99, SMA (focal), and Ki67 was about 40%. Shoulder joint CT and PET-CT showed that no metastasis presented. Germline testing showed decreased copy number of*BRCA1* and *BRCA2*. The diagnosis was cutaneous extraskeletal osteosarcomas of the left shoulder. The patient underwent an enlarged resection, followed by local radiotherapy four cycles. No recurrence or metastasis occurred on a 1-year of follow-up.

**Conclusions:**

Primary cutaneous extraskeletal osteosarcoma (PC-EOS) is rare, and preoperative differential diagnosis is difficult. This is the first report of PC-EOS with decreased copy number of *BRCA1* and *BRCA2*. The presented case highlights the importance of accurate histopathological examination and comprehensive analysis. We considered that *BRCA1* and *BRCA2* genes may are associated with a worse outcome and local recurrence in PC-EOS. But, it may not have been fully recognized.

## 1 Introduction

Extraskeletal osteosarcoma can present as a skin tumor without involvement of deep soft tissues or internal organs. Its clinical presentation is variable and can manifest as subcutaneous nodules or exogenous masses. Due to the lack of specific features, preoperative diagnosis can be challenging. Now we summarize the diagnosis and treatment process of a 28-year-old male with osteosarcoma of the left shoulder, in order to share our experience and lessons learned and improve understanding of this disease.

## 2 Case description

**Figure 1:**
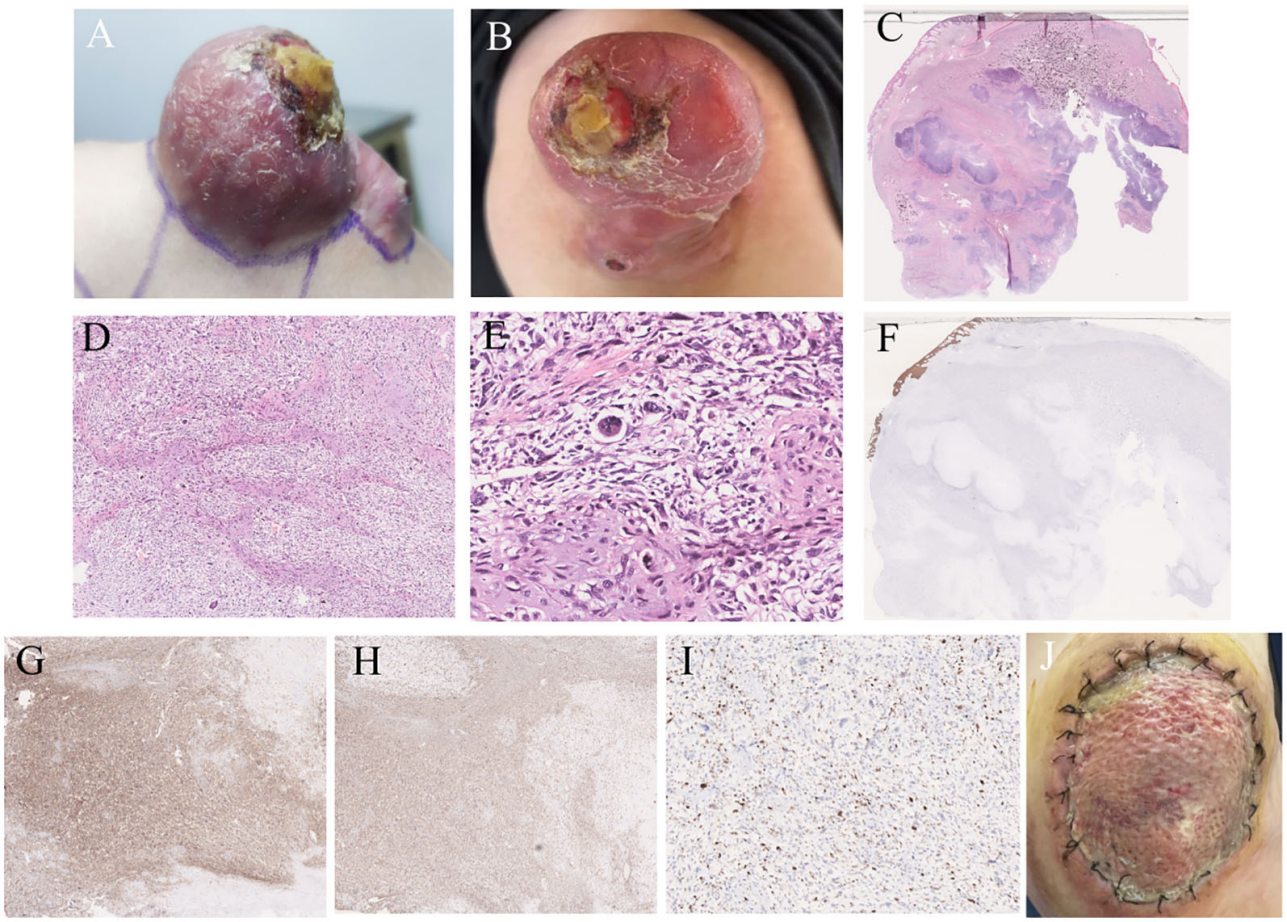
Clinical and histopathological features. **(A)** A 6*6.5*2 (cm) hemispherical mass was presented on the left shoulder. **(B)** scattered scales were visible in the surrounding area, with ulcerated surfaces and yellow crusts on top. **(C)** A large irregular mass was seen in the subcutaneous soft tissue,with erosion in the superficial regions (HE staining, ×4). **(D)** Pleomorphic chondrocytes were visible, with a cell-rich periphery of lobules mixed with areas of osteoblastic activity (HE staining, ×50). **(E)** The tumor cells exhibited pleomorphism with various shapes including spindle-shaped, triangular, and oval. A large number of tumor giant cells and fibroblasts were seen, and nuclear division was easily observed (HE staining, ×200). **(F)** CK (-). **(G)** CD99 (+). **(H)** Vim (+). **(I)** Ki67 (40%+). **(J)** There was a circular incision on the left shoulder measuring approximately 10cm in diameter. And the incision edges were aligned and sutured in place, with mild redness, swelling, and oozing.

**Figure 2:**
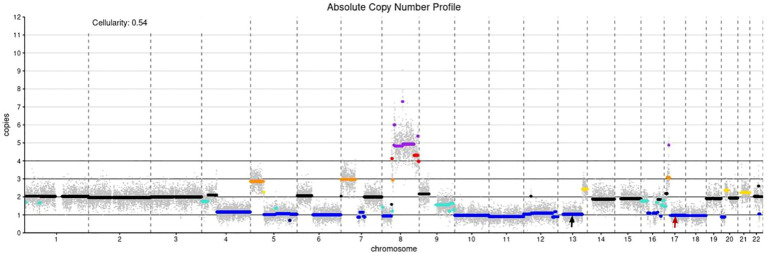
Absolute copy number profile: The color code in the plot is as follows (in parentheses the call in the data frame): black - not called (0); gold - subclonal gain (0.5); turquoise - subclonal loss (-0.5);dark orange - single copy gain (1);blue - single copy loss (-1); red - double copy gain (2); dark blue - double copy or full loss(-2); purple - amplification (3); Simple annotation of copy numbers of coding genes using devtools:*BRCA1* CN= 1; *BRCA2* CN= 1; *MYC* CN=4; black arrow: *BRCA2*, red arrow: *BRCA1.*.

A 28-year-old man presented with an exogenous mass on the left shoulder for 3 months, that was about 3×3×4 (mm) in initial and quickly increased in size over the past time. He had a history of keloids but denied any history of local trauma, radiotherapy, or chemotherapy. Family history was negative for relevant malignancies. Physical examination showed a mass of about 6×6.5×2 (cm) on the left shoulder. (**Figures 1A, B**). The ultrasound examination revealed a hypoechoic solid mass in the subcutaneous tissue that had irregular bordersand uneven internal echogenicity. His results on complete blood cell count and differential count, four coagulation tests, liver and kidney function tests, electrolyte profiles and abdominal ultrasound were negative for disease. The lesion was completely excised. The histological examination showed numerous tumor giant cells and fibroblasts, and nuclear division was easily seen. There is also abundant osteoid, chondroid elements, with delicate lace-like bone or wide bone trabeculae. (**Figures 1C–E**). Immunohistochemistry showed positive for CK, EMA, S100, CD34, CK7, Bcl-2, ACTin, and NSE, and negative for Vim, SATB2, CD99, SMA(focal), and Ki67 was about 40% (**Figures 1F–I**). Shoulder joint CT and PET-CT showed that the tumor localized on the skin without involvement of the shoulder joint or humerus, and no metastasis presented. A diagnosis of cutaneous extraskeletal osteosarcomas of the left shoulder was made. Due to the early onset of cancer, the patient conducted germline testing that revealed *BRCA1*, *BRCA2* and *RB1* losses, and amplification of the *MYC* gene (**Figure 2**). Considering a high possibility of recurrence, the patient underwent an enlarged resection (**Figure 1J**), followed by local radiotherapy four cycles. No recurrence or metastasis occurred on a 1-year of follow-up.

## 3 Discussion

Extraskeletal osteosarcoma (EOS), first reported by Wilson in 1941, is a rare malignant soft tissue sarcoma of mesenchymal origin. It accounts for about 1% of all soft tissue sarcomas and about 4% of osteosarcomas, and produces tumor-like bone or cartilage material without obvious attachment to bone or periosteum (1).

Etiopathogenesis remains poorly understood, potential etiologies are proposed to be sun exposure, previous trauma or radiation, burn scars, malignant melanoma, Paget disease of the bone, and germline abnormalities (2). EOS is commonly found in deep tissues such as limbs, trunk, and retroperitoneum, and rarely in solid organs like liver and breast. PC-EOS is exceptionally rarer. The clinical presentation lacks specificity, it may be with or without pain, and may present with ulceration or bleeding (2, 3). Its size range from small nodules to large exophytic masses with slow-growing, however, rapid growing could occur sometimes. Imaging performance lacks specificity, but calcification and ossification are important manifestations. It has been reported that approximately 50% of cases of extraskeletal osteosarcoma exhibit calcification or ossification (4), with eccentric and mature ossification being more common (5). Therefore, when the suspected disease is found clinically, the CT examination should be improved as much as possible to clarify the calcification or ossification in the lesion. Unlike osteosarcoma, PC-EOS primarily affects the elderly. However, its histological features are similar to osteosarcoma, mainly consisting of spindle cells, bone or osteoid-like material, and cartilage tissue (4). Clinical differential diagnoses included squamous cell carcinoma, basal cell carcinoma, Merkel cell carcinoma, dedifferentiated malignant melanoma, simple cyst, neurofibroma, adnexal tumor, lipoma, traumatic myositis ossificans, and metastasis or cutaneous extension of a tumor originating from bone or deep soft tissue (3). Wide surgical excision is the optimal treatment, and postoperative radiotherapy can improve survival rates and delay recurrence (1). Due to the paucity of reported cases, reliable and specific survival data for PC-EOS are scare (3).

*BRCA1* and *BRCA2* genes were discovered in 1994 and 1995, respectively, that their encoding proteins involved in tumor suppression, regulating cell replication, DNA damage repair, and normal cell growth in the human body. Pathogenic germline variants in the *BRCA1/2* genes lead to an increased lifetime risk of breast, ovarian and further less frequently present cancers in women and an increased lifetime risk of breast, prostate and other tumors in men (6). It has been shown that mutations, genomic instability and loss of heterozygosity resulting in BRCA1/2 inactivation occur in 91% and 78% of osteosarcoma, respectively (7). And BRCA1 and BRCA2 are driver genes for osteosarcoma (8). Loss of the BRCA pathway accelerates p53-associated tumor development, possibly without altering the fundamental tumorigenic processes (9). *TP53* and *RB1* losses and *CDKN2A* loss are associated with a worse outcome and local recurrence in EOS (10). Therefore, we considered that *BRCA1* and *BRCA2* genes may play an important role in the occurrence, development, and prognosis of EOS, which has not been fully described in the literature. Thus, it may not have been fully recognized.

The patient is a 28-year-old young man with a rapidly growing mass on the left shoulder, involving the dermis, and with *BRCA1* and *BRCA2* deletion. He denied any history of local trauma. The onset of the disease may be related to deletion in the *BRCA1* and *BRCA2* genes, which led to the inability of cells to effectively repair DNA damage, resulting in genomic instability that promoted the occurrence and progression of osteosarcoma. However, the study is based on a single case, and family members have no history of related cancers and refused to conduct germline testing, which limits the generalizability of the findings.

## 4 Conclusions

Herein, we describe the case of a 28-year-old man diagnosed with the primary cutaneous osteosarcoma with decreased copy number of *BRCA1* and *BRCA2*, based on histological morphology, immunohistochemical examination, and germline testing. This study is the first to report such a case. Although primary cutaneous osteosarcoma is rare, it has been reported in over 20 cases worldwide. Most patients presented with purplish-red solitary exophytic nodules that are firm in texture, with ulcerated surfaces and commonly occurring on the scalp and extremities (2, 11). Therefore, the possibility of this disease should be considered in the rapid growth of exogenous skin lesions, and early identification and treatment are essential for the prognosis of patients.

## Data Availability

The original contributions presented in the study are included in the article/supplementary material. Further inquiries can be directed to the corresponding author/s.
